# Germany’s journey toward 14 Tesla human magnetic resonance

**DOI:** 10.1007/s10334-023-01085-z

**Published:** 2023-04-08

**Authors:** Mark E. Ladd, Harald H. Quick, Oliver Speck, Michael Bock, Arnd Doerfler, Michael Forsting, Jürgen Hennig, Bernd Ittermann, Harald E. Möller, Armin M. Nagel, Thoralf Niendorf, Stefan Remy, Tobias Schaeffter, Klaus Scheffler, Heinz-Peter Schlemmer, Sebastian Schmitter, Laura Schreiber, N. Jon Shah, Tony Stöcker, Michael Uder, Arno Villringer, Nikolaus Weiskopf, Moritz Zaiss, Maxim Zaitsev

**Affiliations:** 1 Medical Physics in Radiology, German Cancer Research Center (DKFZ), Im Neuenheimer Feld 280, 69120 Heidelberg, Germany; 2 Faculty of Medicine, Heidelberg University, Heidelberg, Germany; 3 Faculty of Physics and Astronomy, Heidelberg University, Heidelberg, Germany; 4 Erwin L. Hahn Institute for MRI, University of Duisburg-Essen, Essen, Germany; 5 High-Field and Hybrid MR Imaging, University Hospital Essen, Essen, Germany; 6 Department of Biomedical Magnetic Resonance, Otto von Guericke University Magdeburg, Magdeburg, Germany; 7 German Center for Neurodegenerative Diseases (DZNE), Magdeburg, Germany; 8 Center for Behavioural Brain Sciences, Magdeburg, Germany; 9 Leibniz Institute for Neurobiology (LIN), Magdeburg, Germany; 10 Division of Medical Physics, Department of Diagnostic and Interventional Radiology, Faculty of Medicine, Medical Center, University of Freiburg, Freiburg, Germany; 11 Department of Neuroradiology, University Hospital Erlangen, Friedrich-Alexander University Erlangen-Nuremberg (FAU), Erlangen, Germany; 12 Institute of Diagnostic and Interventional Radiology and Neuroradiology, University Hospital Essen, Essen, Germany; 13 Medical Physics and Metrological Information Technology, Physikalisch-Technische Bundesanstalt (PTB), Berlin, Germany; 14 Methods and Development Group Nuclear Magnetic Resonance, Max Planck Institute for Human Cognitive and Brain Sciences, Leipzig, Germany; 15 Institute of Radiology, University Hospital Erlangen, Friedrich-Alexander University Erlangen-Nuremberg (FAU), Erlangen, Germany; 16 Berlin Ultrahigh Field Facility (B.U.F.F.), Max Delbrück Center for Molecular Medicine in the Helmholtz Association, Berlin, Germany; 17 Magnetic Resonance Center, Max Planck Institute for Biological Cybernetics, Tübingen, Germany; 18 Department of Biomedical Magnetic Resonance, University of Tübingen, Tübingen, Germany; 19 Radiology, German Cancer Research Center (DKFZ), Heidelberg, Germany; 20 Department of Cardiovascular Imaging, Comprehensive Heart Failure Center, University Hospital Würzburg, Würzburg, Germany; 21 Institute of Neuroscience and Medicine – 4, Forschungszentrum Jülich, Jülich, Germany; 22 MR Physics, German Center for Neurodegenerative Diseases (DZNE), Bonn, Germany; 23 Department of Neurology, Max Planck Institute for Human Cognitive and Brain Sciences, Leipzig, Germany; 24 Department of Neurophysics, Max Planck Institute for Human Cognitive and Brain Sciences, Leipzig, Germany; 25 Felix Bloch Institute for Solid State Physics, Faculty of Physics and Earth Sciences, Leipzig University, Leipzig, Germany

**Keywords:** MRI, Ultrahigh field, Extremely high field, 14T, GUFI

## Abstract

Multiple sites within Germany operate human MRI systems with magnetic fields either at 7 Tesla or 9.4 Tesla. In 2013, these sites formed a network to facilitate and harmonize the research being conducted at the different sites and make this technology available to a larger community of researchers and clinicians not only within Germany, but also worldwide. The German Ultrahigh Field Imaging (GUFI) network has defined a strategic goal to establish a 14 Tesla whole-body human MRI system as a national research resource in Germany as the next progression in magnetic field strength. This paper summarizes the history of this initiative, the current status, the motivation for pursuing MR imaging and spectroscopy at such a high magnetic field strength, and the technical and funding challenges involved. It focuses on the scientific and science policy process from the perspective in Germany, and is not intended to be a comprehensive systematic review of the benefits and technical challenges of higher field strengths.

## Current state of UHF

Magnetic field strengths at or above 7 Tesla (T) in human MRI systems are generally referred to as Ultra-High Fields (UHF). By far, the largest segment in this range is at 7 T, where two commercial vendors have now introduced systems that are labeled for clinical use. There are over 100 7 T systems worldwide, with numbers steadily increasing since the first system received clinical CE and FDA approval in 2017 for brain and small joint imaging. There are also a limited number of systems operating at 9.4 T (400 MHz proton resonance frequency), but the focus of this article is on human systems with even higher magnetic field strengths.

The system with the highest magnetic field currently operating and generating images in vivo is the 10.5 T (450 MHz proton resonance frequency) system at the University of Minnesota, which was installed in 2013 but did not receive approval for in vivo imaging until 2017. The magnet was one of the last magnets produced by Agilent Technologies before they exited the magnet market completely and is wound with 433 km of niobium–titanium (NbTi) wire and cooled to below 3 K. It is passively shielded, weighs 110 tons, and has a warm bore of 88 cm, making it suitable for whole-body imaging [1]. The system is only approved for research use, and in vivo results have been presented both in the brain of macaques [2, 3] and humans [4] as well as in the human torso [5, 6].

The currently highest magnetic field human whole-body MRI system was developed and installed at CEA Paris-Saclay, termed the Iseult project [7]. This unique system operates at 11.7 T (500 MHz proton resonance frequency) with a magnet designed and developed by CEA and produced together with Alstom (now General Electric). The project was initiated in 2004 as a French–German cooperation. The magnet reached field in 2018 and produced first images in 2021. At the time of writing, the system is still restricted to ex vivo and phantom measurements. The magnet employs an actively shielded double-pancake design in driven mode at 1500 A using NbTi wire cooled to 1.8 K with superfluid helium. With a warm bore of 90 cm, it is currently the largest 11.7 T MRI magnet worldwide and weighs 132 tons with a length of 5.2 m and a diameter of 5 m [7]. Clearance for first human subject scans is expected in 2023.

Two head-only 11.7 T MRI systems are currently being put into operation at the National Institutes of Health (NIH) in USA and at Gachon Medical University in Incheon, South Korea. The magnet at NIH was originally produced by Agilent but was damaged during a quench event. It was repaired by ASG Superconductors. The magnet in Gachon was completely designed and manufactured by ASG. Although there are minor differences between the two systems, both are wound from NbTi and have similar parameters: 600 km NbTi wire, 66 tons, passive shielding, and 70 cm warm bore [8]. The system at NIH is shielded with 380 tons of iron. Although both magnets have reached field, neither is producing in vivo images at the time of this writing.

## Next step: extremely high field (EHF)

The German Ultrahigh Field Imaging network (GUFI, described below) has set a strategic goal to develop and establish a human whole-body MRI system with an even higher magnetic field strength than 11.7 T, namely 14 T. Due to the high cost as well as technical and scientific challenges involved in such a project, the system is to be operated as a national research resource open to scientists across Germany and beyond. Access will be granted based on scientific criteria and the feasibility of the proposed research.

At magnetic field strengths above 12 T, the superconducting technology used for the construction of the magnet must fundamentally change, as the material properties of NbTi do not support such high magnetic fields. It therefore seems appropriate to introduce a new classification for human MRI systems above 12 T. We therefore refer to magnetic fields at or above 12 T as Extremely High Fields (EHF) in the rest of this article.

## Scientific motivation for 14 T

The detailed motivations for going to higher static magnetic fields based on potential biomedical and neurocognitive applications are beyond the scope of this article, but have been documented in several previous reports and review papers [9–13]. There have also been Special Issues in scientific journals dedicated to these topics [14–16], and the reader is referred to these sources for details. This contribution focuses on the scientific and science policy process from the perspective in Germany. It is not intended to be a comprehensive systematic technical review of the benefits and challenges of higher field strengths, which have been covered in these previous publications. General motivations are briefly summarized below.

**Fig. 1:**
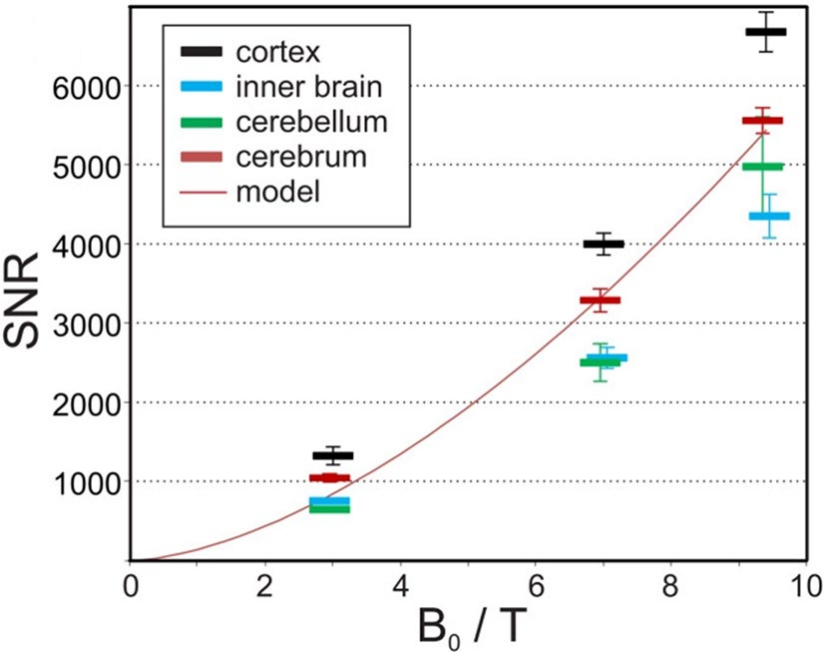
SNR values in four different brain compartments as a function of magnetic field strength B_0_. The red line represents fitting results on the SNR over the entire cerebrum as SNR = *B*_0_^1.65^ (reproduced with permission from Fig. 5b in [17])

The main limitation of MRI is its limited sensitivity due to the low thermal equilibrium magnetization that is governed by the Boltzmann distribution. Higher static magnetic fields provide one path to address this limitation. For most situations involving human imaging, the noise from the sample and not the electronic noise of the receiver is dominant. In this case, the signal-to-noise ratio (SNR) is expected to increase linearly if wavelength effects are ignored, i.e., the quasi-static approximation is made. If the full Maxwell equations are taken into account, theoretical and experimental work has shown a supralinear increase in the UHF and EHF regime. An in vivo comparison between 3, 7, and 9.4 T showed SNR increasing as B_0_^1.65^ over the cerebrum (Fig. 1) [17]. A recent publication comparing experimental measurements of SNR in the center of a spherical phantom at 3, 7, 9.4, 10.5, and 11.7 T showed SNR growing as *B*_0_^1.94±0.16^ [18]. Simulation studies of ultimate intrinsic SNR indicate that the SNR increase is spatially dependent and increases faster for deeper lying tissues than tissue in the periphery [19–21]. This relation could not be confirmed in the experimental work of Pohmann et al. [17], but their results may have been confounded by the different RF coil designs used at different field strengths. In addition to the SNR increase for unaccelerated imaging, both theoretical [19] and experimental works [22–24] have demonstrated the additional advantages for parallel imaging at higher magnetic fields due to the reduced g-factors at shorter wavelengths.

The enhanced SNR can most obviously be invested into higher spatial resolution for anatomical imaging. Already at 7 T, for example, high-resolution vascular images of the human brain have been generated with isotropic voxel resolutions of 140–150 μm using prospective motion correction [25, 26]. Further motivation to pursue higher magnetic fields is provided by the enhanced sensitivity to magnetic susceptibility differences in the tissue, and this superior tissue contrast is especially important for pathologies involving iron deposition or changes in myelin or hemodynamics. Functional MRI (fMRI) is another prime example of an application that benefits from the enhanced specificity provided by the higher contrast-to-noise ratio (CNR) afforded in detecting changes in susceptibility values, in this case associated with shifts in the concentration of deoxyhemoglobin and oxyhemoglobin in the blood [12]. Spectral separation between individual metabolites is also enhanced at higher magnetic fields. Through the combination of increased SNR and spectral separation, techniques such as Chemical Exchange Saturation Transfer (CEST) imaging and Magnetic Resonance Spectroscopic Imaging (MRSI) should profit disproportionately, which has been demonstrated in comparisons between 3 T, 7 T, 9.4 T, and 21 T, the latter field strength acquired in rats [27].

A 14 T MRI system would represent a doubling in field strength compared to the currently highest clinically approved field strength of 7 T. Conservatively, we expect a potential SNR increase of 3–4 if changes in relaxation times and other effects are ignored. If we take the move from 1.5 to 3 T or from 3 to 7 T as models for what to expect at 14 T compared to 7 T, significant improvements in spatial resolution and sensitivity for proton imaging and spectroscopy can be expected. For other nuclei for which even 7 T can still not deliver adequate SNR for high-resolution applications within the practical constraints of scan times acceptable for clinical research in humans (about 1 h), the potential is even greater. Nuclei such as ^19^F, ^23^Na, ^31^P, ^17^O, ^39^ K, ^35^Cl, ^2^H, or ^13^C are particularly interesting for studying both healthy physiology and pathologies in the brain, abdominal organs, heart, or musculoskeletal system. In the torso, where non-cyclical physiological motion may limit the achievable spatial resolution in ^1^H imaging, these concerns are much less relevant at the lower resolutions targeted for X-nuclei. fMRI in the brain using the blood-oxygenation-level-dependent (BOLD) contrast has historically been the driving application for moves to higher magnetic field strengths. The BOLD contrast itself has been shown to be a complicated function of field strength, but to increase more than linearly under certain conditions [28]. In addition, the microvascular contribution increases more than the signal originating from larger vessels, further improving spatial specificity [29, 30]. The higher intrinsic SNR ranging from linear in the periphery [20] to roughly quadratic in the central regions of the brain [18] indicates that the overall functional imaging contrast will increase substantially with field strength, which has been demonstrated at field strengths available to date [31], and this application will be a logical first step at 14 T. The enhanced sensitivity harbors the potential to bridge the gap between the microscopic scale of cognitive neuroimaging that requires invasive approaches and the macroscopic information provided by non-invasive fMRI. It may finally be possible to resolve the mesoscopic scale corresponding to the brain’s individual functional units consisting of conglomerations of many neurons organized into cortical columns and layers and working together to fulfill specific tasks [32, 33]. MR detection will probably still rely on detecting the vascular response rather than measuring neuronal activity directly, however, and thus be limited by capillary spacing, on the order of 40–50 µm.

No fundamental imaging roadblocks are expected to emerge at 14 T as they did when transitioning above 3 T. Even at 3 T, radiofrequency (RF) wavelength effects started to emerge that hampered imaging in large cross-sections, such as the abdomen and pelvis [34]. Such effects can be addressed using parallel transmission techniques as already demonstrated at UHF [35], so we expect rapid progression of 14 T to research application of general structural, functional, and metabolic MRI techniques in groups of individuals to study healthy physiology, aging, or pathologies.

Neuroscientific, basic physiologic, and even clinical research are expected to strongly benefit from the aforementioned sensitivity and specificity gains. Whether 14 T will ultimately be applied to examine individuals for the purpose of clinical diagnostics is an open question, at least in terms of the time-frame to achieve this transition. Given the current cost for a 14 T magnet that is expected to be on the order of tens of millions of Euros/dollars, the technology would have to demonstrate a significant impact on individual patient outcome to justify the higher costs of the diagnostic test. This impact certainly cannot be excluded in areas, such as, for example, the (differential) diagnosis of neurodegenerative or neuropsychiatric diseases or providing clear biomarkers for personalizing tumor therapy, but these questions can only be answered through research.

A fundamental question to be answered when introducing a new field strength is whether the magnet should enable head-only examinations or be capable of imaging the entire body. In the latter case, the cost of the magnet is significantly higher, since more superconducting material is needed to achieve the larger size, and the technical risks are elevated, as well. However, GUFI has made a clear commitment to pursue a whole-body design to permit the investigation of scientific questions in the brain, trunk, and limbs. This decision was underscored by the results of a survey performed in 2016 within the wider German scientific community, including scientists outside of GUFI. Importantly, this survey was performed targeting not methodological research at 14 T, but rather potential biological and medical questions. The list of project ideas generated by this survey clearly showed a substantial need for investigations outside the brain to target questions involving muscle channelopathies and dystrophies, muscle metabolism, cancer of multiple organs including breast and prostate, liver and heart metabolism, cardiomyopathy and cardiac fibrosis, bone microvascularization, cystic fibrosis, acute kidney injury and chronic kidney disease, and others. In the brain and head, applications included cancer, glaucoma, ocular diseases and disorders of the nervus opticus, vascular pathologies, ataxias, brain metabolism, neurodegeneration, fMRI including layer-specific investigations for multiple brain networks, high-resolution structural atlases, epilepsy, multiple sclerosis, and radiation therapy planning and monitoring. Studies requiring whole-body imaging capability made up over 40% of the submitted proposals. Even for many neuro applications, imaging of the spine below the head–neck region is often required.

The choice to pursue 14 T is also significant because of the requisite disruption in superconducting technology at this field strength. Today’s human MRI magnets are constructed with NbTi superconductor and are approaching the limits of that material. 14 T requires a move to a different superconducting wire, and this comes with substantial technological risk. Once this barrier is broken through, ample new leeway exists for large bore diameters, even higher magnetic fields in the future, and open magnet geometries suitable for MR-guided interventions and radiotherapy. These technological developments could also have important consequences for other scientific fields that require large-size magnets.

## GUFI

The German Ultrahigh Field Imaging (GUFI) network was founded at the end of 2013 with the aid of German Research Foundation (DFG) funding provided by a call to establish Core Facilities including distributed national networks, with GUFI as an early example. The GUFI network now includes centers from 11 German cities who perform human UHF MR imaging or spectroscopy at magnetic field strengths of 7 and 9.4 T [36]. Several international partners are also affiliated with the network. The first funding period ended in 2016 and was extended for a second period from 2017 to 2021. Since the conclusion of DFG funding, the network members have continued their joint efforts to promote clinical and scientific UHF technology using internal resources.

The overall goal of GUFI is to facilitate and harmonize the work of the German UHF MR sites and make the expensive and highly complex UHF MR technology accessible to a wider range of researchers and clinicians. The focus of the first funding period was to promote communication between the centers via a web-based portal as well as regular face-to-face meetings. A consensus guideline document was formulated giving basic recommendations regarding access procedures and rules for UHF MR research infrastructure [37]. A consensus recommendation regarding safety of subjects with passive implants at UHF MR was also issued by the GUFI members [38]. The network established quality assurance procedures and a corresponding measurement phantom suitable for monitoring system performance and ensuring that each UHF MR system is working at peak performance [39, 40]. As an extension of this work, GUFI conducted and published a study examining in vivo measurement reproducibility across 7 T sites. After an international multicenter research study of neurochemical profiles at UHF [41], the GUFI study was the first study to pave the way for multicenter research studies utilizing structural imaging at UHF [42].

In the second funding period, GUFI continued the networking and communication activities. Consensus safety procedures, including staff and user training based on an online MR safety course, were established and a database of specific implants and accessories that can be safely accepted for imaging at UHF was compiled. GUFI also reached consensus regarding testing and evaluation of self-built radiofrequency coils contributing to a White Paper on experimental RF hardware as part of a working group of the International Society for Magnetic Resonance in Medicine [43]. A second multicenter trial was conducted expanding knowledge on reproducibility to quantitative imaging methods [44].

## Euro-BioImaging

In several aspects, the European Euro-BioImaging initiative was a precursor and model for the GUFI activities. Euro-BioImaging was designed to provide transnational user access to large-scale biological and biomedical imaging research infrastructure. From 2010 to 2014, the European Strategy Forum on Research Infrastructures (ESFRI) funded the preparatory phase of Euro-BioImaging to establish the full concept, identify partner countries and infrastructure providers, and prepare the legal framework and contracts. From the beginning, the German research community in microscopy and medical imaging were intensively involved, with UHF MR as part of medical imaging. After an interim phase, Euro-BioImaging was successfully established as a legal entity (ERIC—European Research Infrastructure Consortium) in 2019 with a central hub in Turku, Finland, the European Molecular Biology Laboratory (EMBL) in Heidelberg, Germany as the biological imaging hub, and Turin, Italy as the biomedical imaging hub. Euro-BioImaging comprises 16 members and one observer. European member states and EMBL (an intergovernmental research organization [45]) are members of Euro-BioImaging. The mission of Euro-BioImaging is to provide access, services, and training for state-of-the-art imaging technologies for all life scientists in Europe and beyond. With 33 nodes and 148 imaging facilities, Euro-BioImaging provides access to 31 biological and 16 biomedical imaging technologies to support over 100 user projects per year [46].

To actively participate and contribute to Euro-BioImaging, a national consortium including the German UHF MR centers applied to an open call for large research infrastructure projects for the German national roadmap issued by the German Federal Ministry of Education and Research (BMBF) in 2012. The application proposed inclusion of German Euro-BioImaging as the German contribution to Euro-BioImaging on the national ESFRI roadmap. The medical imaging contribution included UHF MR, MR/PET, and phase-contrast X-ray imaging. In 2013, the scientific evaluation by the German Council of Science and Humanities (Wissenschaftsrat) scored German Euro-BioImaging highest among 13 national research infrastructure proposals [47]. Following further economic and political evaluation, the BMBF ultimately decided to put three competing projects on the national roadmap. Germany has not yet become a member of Euro-BioImaging.

## Toward funding the 14 T

### National Biomedical Imaging Facility (NIF)

In 2015, the German Federal Ministry of Education and Research (BMBF) again issued an open-topic call for project proposals to establish research infrastructures of national importance with a submission deadline in January 2016 (as an update of the 2012 call). The GUFI consortium participated in a proposal coordinated by the Forschungszentrum Jülich and the German Cancer Research Center (DKFZ) to establish a coordinated, open National Biomedical Imaging Facility (NIF) for research and application of technologies including MRI, PET, MEG, EEG, and hybrid MR/PET [48]. The focus of NIF was on multimodal imaging, motivated by the desire to acquire complementary datasets enabling comprehensive neuroscientific/biomedical investigations. A centerpiece of the strategy of NIF was the establishment of a whole-body 14 T MRI system, under the aegis of GUFI, with the perspective to progress to an even higher field strength of 20 T after the end of an initial 10-year project duration. The GUFI community recognized that a concerted, national effort was needed to tackle the development of a 14 T system, and all sites agreed to contribute technology to the effort. A main aim of the proposal was to ensure that Germany's universities, research institutions, and industry could maintain their pioneering role in research and commercial application in the field of UHF and EHF MRI.

As part of the consultations, a workshop was conducted in Cologne in 2016 to which researchers from across Germany were invited and at which possible applications of 14 T were identified and discussed; in advance of the workshop, specific research proposals at 14 T were solicited. The workshop outcome underlined the importance of research across all organ systems of the body and reinforced the conviction that a whole-body system should be targeted. The concept was evaluated by the German Council of Science and Humanities (Wissenschaftsrat) and received the highest scientific rating of all 12 applications submitted [49] across different scientific disciplines. After the BMBF's social and political prioritization, the project was not selected for funding, as announced in 2019.

### Major instrumentation initiative for 14 T

In 2020, the GUFI network submitted a concept to the DFG proposing that a call be issued to fund a human 14 T MRI for biomedical research as part of a Major Instrumentation Initiative. Since the original proposal to the BMBF submitted in 2016, many of the technical and economic uncertainties regarding the magnet had been addressed by potential suppliers, and the overall project costs had dropped substantially, but still on the order of 10 s of millions depending on the exact magnet specifications. Although the scientific reviews of the proposal were very positive and the project was strongly recommended for further development and implementation, the DFG could not fund such a system with the resources available in its funding programs.

Since the BMBF and DFG represent the two main sources of public research funding in Germany, funding of a 14 T MRI system currently remains unclear. As the number of projects worldwide involving systems with magnetic field strength at or above 10.5 T increases, there is growing motivation to support this international development by establishing such a national resource in Germany as well, and GUFI researchers continue to pursue alternative funding avenues for this project.

## 14 T MRI design considerations

### Magnet

**Fig. 2:**
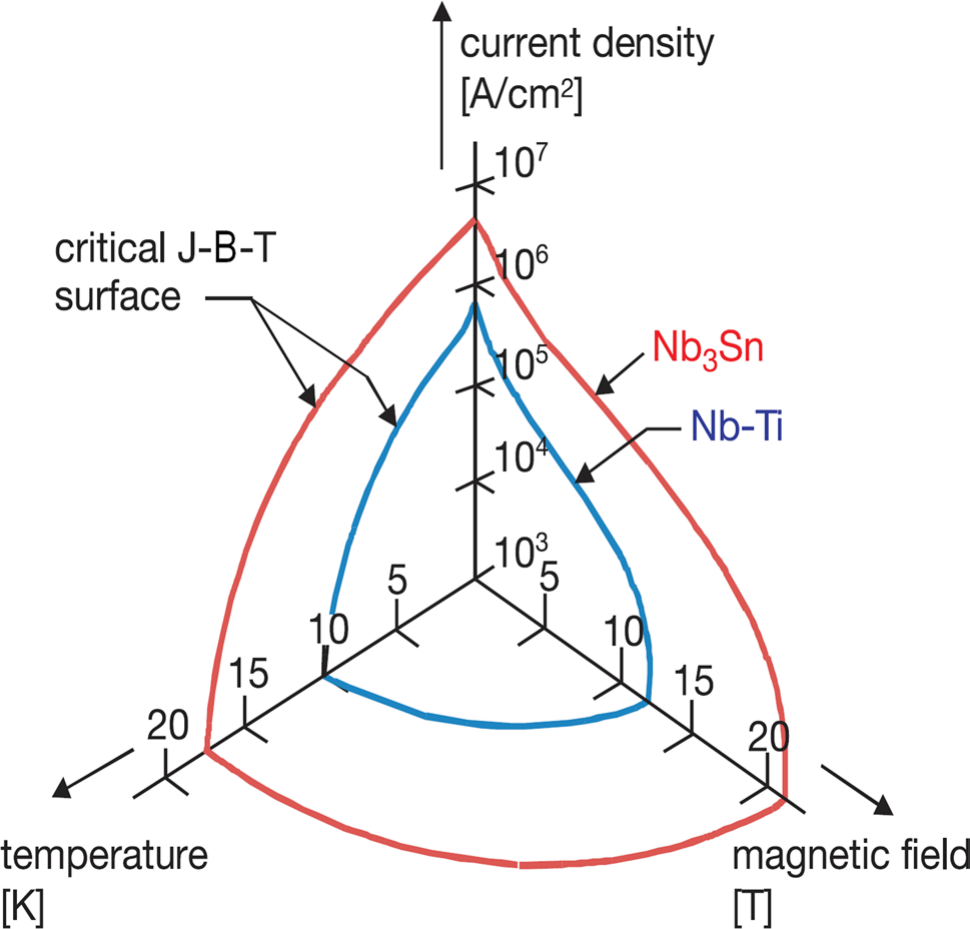
The critical surfaces for NbTi and Nb_3_Sn showing the parameter volume under the critical surface in which the material is superconducting. Higher magnetic fields B can be achieved to a certain extent by lowering the current density J in the superconductor or reducing the temperature T. Above about 12 T, NbTi is no longer viable for human MRI magnets (adapted and reproduced with permission from [50])

Superconductors can be characterized by a critical surface giving the maximum current density J that is supported by the material as a function of the temperature T and magnetic flux density B at which the superconductor should operate (Fig. 2). A further parameter influencing the superconducting property is mechanical strain, but this parameter is ignored in generating the critical surfaces of Fig. 2.

Up to 12 T, NbTi is the superconductor of choice, since its material properties allow it to generate the required magnetic field, it is relatively easy to manufacture and wind into the required geometry, and it provides a cost-effective solution compared to alternatives. At 11.7 T, however, the operating point of NbTi is at the material limits, and additional measures must be taken to ensure superconductivity. The Iseult/Inumac 11.7 T magnet is, for example, cooled to 1.8 K [51] rather than the 4.2 K liquid helium temperature used in most conventional NbTi magnet designs. The limit of NbTi at conventional 4.2 K is around 10 T [52]. The 10.5 T system in Minnesota is operated at 2.3 K to provide a safety margin to the critical temperature of 2.8 K [1].

At 12 T and above, alternative superconducting materials include niobium-tin (Nb_3_Sn) and also multiple high-temperature superconductors (HTS). These materials are significantly more expensive than NbTi, generally by at least an order of magnitude. However, because they have superior material characteristics, i.e., higher critical current density, critical temperature, and/or critical magnetic field and thus larger usable parameter space under the critical surface (see Fig. 2), less wire material can be used in winding the magnet, so that the cost of the magnet does not scale by this wire cost factor directly. Nb_3_Sn does have the disadvantage, though, of being mechanically brittle after it is reacted, making magnet construction and repairs extremely challenging. HTS materials such as DI-BSSCO (Bi2223) are also mechanically brittle. However, the mechanical properties can be improved by reinforcing them with stainless steel, copper, or nickel alloy tapes [53]. In the case of nickel alloy, the critical double bending diameter can be halved from 80 to 40 mm [53]. The critical temperature T_c_ of the DI-BSSCO material is roughly 110 K [54], and commercially available tapes have a critical current I_c_ around 180 A in their self-field at 77 K [53]. An additional advantage of HTS materials is that the energy depositions from gradient/magnet interactions are less likely to provoke a quench, since the safety margin between magnet operating temperature and superconductor critical temperature will likely be quite large at 14 T.

Another strategy to optimize cost is to utilize NbTi in those areas of the magnet with fields below approximately 10 T and reserve the more expensive material such as Nb_3_Sn for the inner cylinder of the magnet with the highest magnetic fields. This design strategy was followed for the 21.1 T system in Tallahassee, Florida, which is currently the highest field strength available for preclinical imaging [55].

Another challenge is that a human-sized magnet will need to be wound from many individual sections of superconducting wire, because wire materials are only available in limited lengths, necessitating, in general, hundreds of joints between the sections. With NbTi, it is possible to produce reliable superconducting joints [56]; however, this is extremely challenging in the alternative superconductors. Due to the residual resistance of the multiple joints in the magnet, most EHF human magnet designs do not operate in the persistent mode, rather they are connected to a power supply (driven mode) that overcomes the small but finite residual resistance of the joints. The power supply must be extremely stable to avoid field drift.

A further design decision is whether the magnet should be actively or passively shielded. Most clinical MRI systems nowadays have counter-wound coils outside the main magnet coils that counteract the stray magnetic field around the magnet and reduce its magnetic footprint. This allows siting the magnets in smaller rooms and avoids the requirement to restrict access to an excessively large area around the magnet room itself. Additionally, it reduces the weight and cost of the iron cage needed for passive shielding, as discussed below. On the other hand, active shielding complicates the design of the magnet and increases the magnet’s own cost, size, and weight because of the additional superconducting material needed for the shielding coils. In addition, the peak magnetic field inside the magnet superconductors is higher than a non-shielded design. Active shielding was chosen for the Iseult/Inumac 11.7 T human magnet, which has a cryostat outer diameter of 5 m to accommodate the additional coils [57]. As an alternative to active shielding, passive shielding with iron can be pursued. This solution puts higher demands on the building that houses the system and requires a larger siting area. In the case of the 11.7 T Iseult/Inumac system, a passive shield would have required at least 800 tons of iron [51]. Nevertheless, passive shielding reduces the risk of novel magnet designs, and the first generation of 7 T human MRI magnets were passively shielded. The weight (and cost) of the passive shield can be lowered or even eliminated if enough area can be reserved around the magnet. Such a non-shielded design may require dedicated local shielding of the system electronics and other equipment needed to operate the system, and the controlled access area will have to be accordingly large to prevent unwanted interactions with magnetic materials.

A final magnet design choice that should be mentioned is the winding geometry. Most human MRI magnets follow a solenoidal winding pattern. The most straightforward design is a long solenoid with additional compensation coils outside the main solenoid to correct for the field inhomogeneity introduced by the finite length of the main solenoid [58]. Alternatively, many modern clinical MRI systems use discrete coil packages to replace the main solenoid. This approach is more cost-effective and allows the magnet to be shorter and more compact, however, at the cost of a higher peak field inside the magnet. The peak field inside a modern, compact 3 T magnet may be around 6 T [58]. A third option is to adopt a winding geometry often found in high-energy physics or fusion applications called a double pancake. In this geometry, each double pancake is wound from the outside diameter to the inside diameter of the pancake and then from the inside to the outside, thus resulting in the “double” layer. This approach is highly modular, since the magnet consists of a stack of such double pancakes, each of which can be individually replaced, but there are challenges to ensuring adequate homogeneity. The Iseult/Inumac 11.7 T main magnet consists of a stack of 170 double pancakes [57].

Given the enormous challenges involved in designing and constructing a 14 T whole-body or even head-only magnet, an important question is which company or organization would be suitable to take on this challenge. The most straightforward solution would be to source the magnet from a single vendor who provides both the design and manufacturing capability. However, in many projects in high-energy physics or fusion where powerful magnets are needed, a common approach is to separate the design and construction between two different entities. In some instances, an academic laboratory provides the design and an industrial partner provides the manufacturing capability. In the case of a 14 T magnet, organizations that might be considered include MRI system vendors, such as GE, Philips, and Siemens, who have all provided 7 T human MRI systems; however, the magnets of all of the initial 7 T systems were sourced from third parties, although Siemens now manufactures its own 7 T magnets. In terms of academic laboratories, CEA in France, which worked on the design of the 11.7 T Iseult/Inumac magnet, the National High Magnetic Field Laboratory in the U.S., or the Chinese Academy of Sciences are candidates. From the field of high-energy physics, companies focused on one-of-a-kind magnet solutions such as Tesla Engineering Limited, ASG Superconductors, or Bilfinger Noell can also be considered.

Some of the aforementioned organizations have expressed interest in participating in a 14 T project and have generated concepts for magnet designs. However, very little is available in the scientific literature. ASG Superconductors has published very rough design parameters for a 14 T head-only system on their web pages (14 T, 70 cm warm bore, < 150 tons weight) [8], but no further details are currently public. Recently, at least two concepts have been published for 14 T human magnet designs in the scientific literature. The first originates from Neoscan Solutions in Germany [59]. The second comes from the Chinese Academy of Sciences [60]. The two designs are quite different in terms of the underlying design decisions that were made. In the case of the Neoscan magnet, the superconductor is an HTS (Bi2223), which is used for all of the windings. The magnet is wound as a stack of double pancakes, is passively shielded (although the stray field is less than the first generation of passively shielded 7 T whole-body magnets that were wound as compensated solenoids), and requires a stabilized power supply, since it is not persistent. Due to the high critical current that can be supported even at 14 T, wire consumption is only 455 km with a weight of around 8.5 tons, and the magnet is extremely compact, with a length of 1.9 m and a diameter of 1.3 m, even though the warm bore of the magnet is 80 cm (minimum superconducting coil inner diameter 86 cm). The magnet would thus be smaller and lighter than current 7 T designs based on NbTi and in fact comparable to the dimensions of the early human 3 T MRI magnets.

In the case of the design from the Chinese Academy of Sciences, compensated solenoids are investigated. The design employs active shielding, and Nb_3_Sn is used for the inner coil layers where the magnetic field is higher, whereas NbTi is used for the outer layers including the shield coils. The homogeneous imaging region is 50 cm in diameter. Wire consumption is 253 km of Nb_3_Sn and 1567 km of NbTi. The magnet has a length of approximately 3.4 m and a diameter of about 3.56 m. Although the warm bore of the design is not explicitly stated, the inner diameter of the superconducting coil windings is 96 cm, indicating that the warm bore is probably about 90 cm [60].

Even though the two 14 T designs do not allow direct comparison, they do give many hints at the design trade-offs and indicate what is possible using different superconductor materials. Both designs open new technological pathways, which is also relevant for lower field strengths and other applications. Table 1 summarizes important design parameters of various magnets with field strengths ≥ 10.5 T for comparison.

**Table 1:** Summary of magnet design parameters for human MRI systems ≥ 10.5 T (tbd = to be determined)

Parameter	Magnet						
	10.5 T Minnesota [1] and personal comm	11.7 T Iseult [7] and personal comm	11.7 T NIH [58] and personal comm	11. 7 T Gachon [8] and personal comm	11.7 T UK [61] and personal comm	14 T Chinese Academy of Sciences [60]	14 T Neoscan Solutions [59] and personal comm
Warm bore	88 cm	90 cm	68 cm	70 cm	83 cm	90 cm	80 cm
Shielding	Passive (600 t)	Active (no additional passive shield)	Passive (380 t)	Passive (600 t)	Passive (~ 500 t)	Active	Passive
5-Gauss contour without passive shield (radial x axial)	Unknown (6.3 m × 12.5 m with passive shield)	10.5 m × 13.5 m	21.4 m × 27.0 m	Unknown (approx. 21.4 m × 27.0 m)	tbd	8 m × 10 m	16 m × 21 m
Superconductor material	NbTi	NbTi	NbTi	NbTi	NbTi	NbTi and Nb_3_Sn	DI-BSCCO (Bi2223)
Winding geometry	Solenoidal	170 Double pancakes	Solenoidal	Solenoidal	Solenoidal	Solenoidal	177 Double pancakes
Conductor length or weight	433 km	220 km (160 km main coil, 60 km two shield coils)/64 t	572 km	600 km	tbd	1567 km NbTi / 253 km Nb_3_Sn	455 km / 8.5 t
Size (L x W)	4.1 m × 3.2 m	5.2 m × 5.0 m	3.7 m × 2.7 m	3.7 m × 2.7 m	tbd	3.4 m × 3.6 m (Without cryostat)	1.9 m × 1.3 m (Without cryostat)
Weight	110 t	132 t	60 t	62 t	tbd	Unknown	15 t
Operating temperature	2.3 K	1.8 K (Superfluid)	2.5 K	2.5 K	2.2 K	Unknown	4 K
Stored energy	280 MJ	338 MJ	194 MJ	200 MJ	tbd	551 MJ	133 MJ

### Safety and bioeffects

MR is considered non-invasive and “safe” if the limits regarding electromagnetic exposure are observed and good practice is employed in the exclusion of subjects with contraindications (e.g., non-suitable metallic or electronic implants). Currently, European and international guidelines (EU Directive, ICNIRP, FDA, and SCENIHR) consider exposure to static magnetic fields up to 7 or 8 T as no significant risk for human subjects. 14 T MRI will be significantly above these current guidelines, and safe operation has to be established, while exposure to RF and gradients can stay within the current guidelines.

**Fig. 3:**
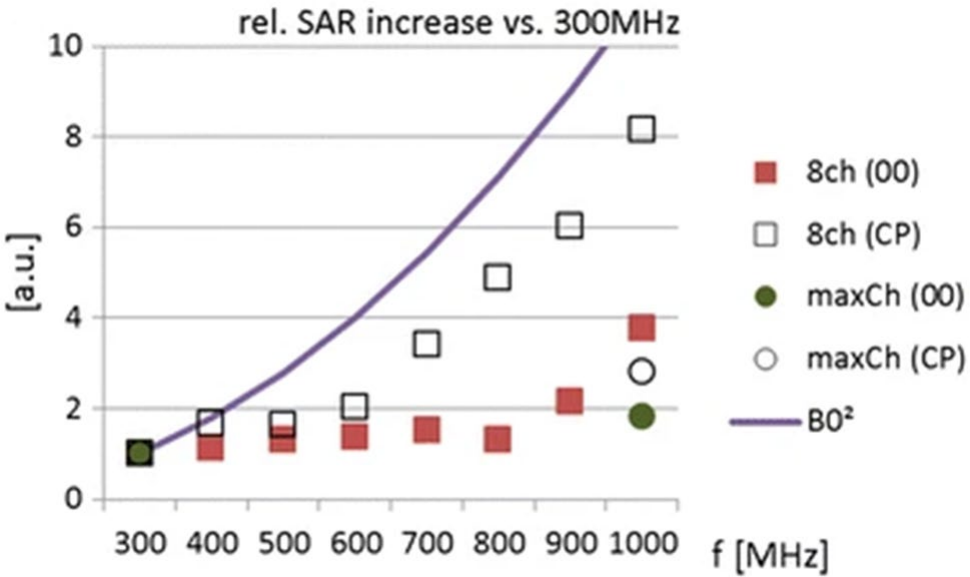
Relative 1-g local SAR increase versus 300 MHz for an 8-channel dipole array driven with all elements in phase (00) or with circular polarization (CP). The maxCh array has 8 elements at 300 MHz and 19 elements at 1000 MHz (reproduced with permission from Fig. 5 g in [63])

RF Specific Absorption Rate (SAR) levels are generally expected to increase with field strength, but in contrast to earlier predictions that did not take the full Maxwell equations into account, theoretical work has shown that the ultimate intrinsic global SAR level is expected to flatten out at higher field strengths rather than increasing quadratically [62]. At UHF, the local SAR limit is typically reached before the global SAR limit, at least for large objects, and must also be considered. Simulations in a human brain model based on physically realizable RF coils at 23.5 T indicate local SAR increases of 1.3–2.7 vs. 7 T if the number of channels is increased to exploit the decreasing inter-element coupling at higher frequencies (Fig. 3) [63], whereas another study showed a whole-head SAR increase by a factor of 1.7 between 7 and 14 T [64]. Work using MR-based simulation pipelines at 890 MHz (corresponding to the resonant frequency of ^1^H at 20.9 T) in various human head models for mobile phone antennas has demonstrated that the fundamental simulation tools to perform efficient B_1_^+^ and SAR calculations are in place even at these high frequencies and emphasize the need for careful consideration of the human tissue model utilized [65]. Nevertheless, SAR will remain a major challenge at EHF, with many questions to be answered. Recent work at 10.5 T, for example, indicates that SAR variations in body regions with respiratory movement are increased compared to 7 T [66].

Mechanical forces and torque are not critical in (mostly) diamagnetic or weakly paramagnetic human tissue. Lorentz forces on moving charges, however, including flowing blood, have been of some concern for very high field strength. The vestibular system is also affected when subjects move into or within a strong magnetic field, leading to side-effects such as vertigo. The largest study of such effects has been conducted by the German UHF community, and in more than 3000 subjects at 7 T and 9.4 T, only minor and transient effects have been reported [67]. Several other studies showed a good tolerance of 7 T examinations by human subjects [68–70]. Pharmacologic intervention (diphenhydramine) or even placebo can reduce the incidence of vertigo by up to 50%, potentially allowing for an increase in field strength even if the effects increase [71].

A recent study in mice exposed for a single session of 60 min to 16 T static as well as 700 MHz RF fields found no severe effects. During the exposure, the mice were restrained from movement inside a 50 mL centrifuge tube. The researchers did observe tight circling immediately after exposure, but no tight circling behavior was observed 7 or 14 days post-exposure [72]. Another study exposed mice to static magnetic fields between 3.5 and 23.0 T for 2 h [73]. Each mouse was confined to a small chamber 38.5 mm in diameter × 80 mm long. They found no long-term negative effects on mouse locomotion, anxiety level, social behavior, or memory, and that in fact the short-term exposure to static magnetic fields might even have beneficial effects on mouse mood and memory. They did, however, observe a transient reduction in the sense of balance and reduction in locomotor activity, although they did not report circling. A further study performed in mice with extensive and repeated exposure to 16.4 T revealed that the mice continued to circle in a preferred direction up to several weeks after exposure cessation [74], indicating long-term changes in motor coordination and balance most probably related to interactions with the vestibular system. A separate cohort exposed to 10.5 T did not show these effects. In this study, the mice were placed pairwise in plastic bins (8 cm H × 7 cm W × 14 cm L) and free to move inside the magnetic field, in contrast to the situation during human MRI exams during which the subjects normally do not move. Further animal studies are required to verify and understand the underlying neural mechanisms of the observed behavior (e.g., is the peripheral or the central vestibular system affected) and to clarify whether the impairment is of transient or permanent nature. Ultimately, even transient physiological side-effects may place a limit on the highest magnetic field to which subjects are willing to voluntarily expose themselves.

Recently, potential cellular and molecular effects of MR exposure arising already at 1.5 T have raised attention. In particular, the observation of double-strand breaks (DSB) has been reported in non-controlled studies [75–78]. A more recent review summarized the controversial data and identified several gaps in knowledge [79]. Furthermore, possible genetic damage in the form of DSBs and micronuclei formation was investigated with the most sensitive immunologic methods at the highest possible human exposure to magnetic fields, gradients, and RF within the current guidelines, and no increased DSBs in ex vivo blood samples nor after in vivo exposure was detected [80, 81].

No human subject exposure studies are yet available at magnetic field strengths of more than 10.5 T, and no significant effects were observed at that field strength [82]. Currently, MRI projects up to 11.7 T are ongoing, but safety has yet to be demonstrated to receive human subject approval. Human subject safety is therefore a prerequisite and a potential risk for the establishment of MRI at 14 T. Apart from technical and methodological challenges, the establishment of safe operating procedures and detailed evaluation of possible bioeffects due to electromagnetic fields are therefore important motivators for a stepwise approach to reach the highest MRI field for human application.

### Magnetic field gradients

A major challenge for EHF MRI relates to the magnetic field gradient and shim systems. Conventional MRI relies on gradient systems producing linear gradient fields over the full imaging field-of-view. The use of traditional gradients at EHF will pose challenges with respect to mechanical forces and acoustic noise [83, 84]. The problem is exacerbated by the fact that higher fields require gradients of higher performance to achieve comparable image quality with respect to magnetic field inhomogeneities, chemical shift artifacts, faster transverse relaxation, and physiological noise, all of which scale with field strength. Early 7 T systems improved their research potential significantly with the introduction of stronger whole-body gradient sets in the second-generation systems. Recently, very-high-performance gradient systems have been presented by major MRI vendors, albeit at lower magnetic field strengths. General Electric has introduced the MAGNUS head-only gradient at 3 T with 200 mT/m and 500 T/m/s [85]. Siemens has installed a similar gradient, termed Impulse, at 7 T with 200 mT/m and 900 T/m/s, and Philips presented experimental gradients with 100 mT/m and 1200 T/m/s at 3 T [86] or up to 5000 T/m/s at 7 T when driving in supersonic resonance [87, 88]. All of these systems have smaller bore sizes than required for whole-body imaging. For 3 T, very powerful whole-body gradients have been designed in the context of the Human Connectome Project with 300 mT/m and a more conventional slew rate of 200 T/m/s [89]. Plans for a Connectome 2.0 gradient with 500 mT/m and 600 T/m/s with 44-cm bore size at 3 T have been published [90]. Application of any of these gradients or similar designs to 14 T will remain a major engineering task and need to consider electromagnetic and mechanical interaction between the gradient system and the magnet [91]. To extract the maximum in performance from a 14 T MRI system, the potential for fast and high-resolution imaging (while reducing TE) and encoding capability (e.g., for diffusion encoding) should be maximized by employing the strongest and fastest gradient sets available.

An alternative approach to conventional (linear) gradients is the use of non-linear, generally non-bijective, encoding fields [92], by allowing the spatial encoding to be performed with a combination of local gradient coils. Such non-linear spatial encoding fields necessitate the use of special reconstruction algorithms [93]. By generating local gradients only within the target area, peripheral nerve stimulation as well as Lorentz forces on the gradient coil elements and therefore acoustic noise can be considerably reduced. Such non-linear gradient systems can be realized as multi-coil arrays that consist of many small coils of low inductance and thus allow for much faster switching at moderate driving voltages, as demonstrated recently with an 84-channel head-sized matrix gradient coil at 3 T [94]. Their disadvantages in varying spatial encoding capability need to be considered, and combinations with linear gradients (either through additional hardware or different combinations of multi-coil fields) may in part address this shortcoming.

Multi-coil gradient systems also bridge the gap to approaches for high-order shimming. Current concepts using global, static shim systems based on spherical harmonics are difficult to extend to very high orders and are challenging due to the increase in current required to drive the compensation fields, in particular outside the brain [95]. Alternative approaches including shim array coils may be a solution [96] and are similar in concept to the non-linear multi-coil gradient arrays. For instance, the above-mentioned 84-channel matrix gradient coil is suitable for both spatial encoding and local high-order shimming [94]. Novel shimming concepts adapted to the human anatomy have been proposed recently [97, 98] that are particularly advantageous at UHF and beyond [99].

A 14 T EHF MRI system will likely start with one or two replaceable conventional high-performance gradient systems of head and whole-body size. It will then become a testbed for more advanced approaches.

### Parallel RF transmit and SAR supervision

The physics of EHF at 14 T also puts high demands and challenges on the RF excitation of the MR experiment. The RF wavelength decreases with increasing magnetic field strength. While the RF excitation wavelength of ^1^H nuclei in water-like human tissue is approximately 12.9 cm at 7 T (300 MHz Larmor frequency), it is 10.1 cm at 9.4 T (400 MHz) and around 8.2 cm at 11.7 T (500 MHz) [100]. Consequently, at 14 T (600 MHz), the RF excitation wavelength of about 7.2 cm will be much shorter than the cross section of any brain or body region to be imaged. It is well known from UHF MRI that this condition leads to an RF distribution that is non-uniform due to destructive and constructive interferences and thus to spatially inhomogeneous RF excitation, with the consequence that the image contrast shows spatial variations and signal voids, and, furthermore, safety is impacted, since the distribution of the RF electric fields is also affected. These fundamental challenges of RF signal excitation at EHF call for new and innovative developments in multichannel RF excitation, homogenization of B_1_, RF energy supervision, and RF coil design.

A key solution to these challenges at UHF has been the introduction of parallel RF transmit (pTx) techniques [35, 101–108]. Here, the RF excitation is performed by multiple independent Tx channels that are connected to RF coils with multiple independent transmit RF elements. Theoretically, each Tx channel and Tx coil element can excite the sample with an individual RF pulse form, amplitude, and phase of the RF signal, providing high degrees of freedom and an effective means to homogenize the RF excitation in UHF brain and body MRI [35]. It has been demonstrated that an increasing number of independent RF channels in a pTx setup provides higher flexibility and, thus, improved flip angle homogenization even in challenging body regions [109]. A pTx system and associated RF transmit coils with high channel count can be considered a technical precondition for successful RF excitation in the human brain and body at EHF, but with the need to increase the number of channels vs. UHF to achieve sufficient degrees of freedom.

Simulation work in the human head at multiple frequencies indicates that MRI up to 23.5 T (1 GHz) should be technically feasible [63]. For an 8-channel dipole array, the increase in local 1-g SAR at 14 T relative to 7 T was roughly 2 for the circularly polarized mode. In the body, numerical simulations at 14 T of an 8-channel fractionated dipole array indicate that sufficient flip angle uniformity in deep-lying regions of interest cannot be obtained with static pTx and 8 channels without incurring very high local SAR values, which implies the need for low duty cycle to comply with SAR limits [110]; these results indicate that for body imaging, > 8 channels and/or dynamic pTx pulses will be necessary at 14 T to enable practical imaging applications. Experimentally, good results in the body have been obtained at 10.5 T utilizing a 10-channel dipole array with static and dynamic pTx [5, 111]. Extrapolating to 14 T, 16 channels appear to be a reasonable starting point for first research studies in the body. With this in mind, electromagnetic simulations have examined B_1_^+^ uniformity and efficiency challenges of cardiac MRI at 14 T using a 16-channel array of fractionated dipole antennas. Static pTx shimming was found to be no longer sufficient to overcome B_1_^+^ inhomogeneities. The simulations demonstrated that kT-point pTx pulses [107] are highly suitable for mitigating B_1_^+^ inhomogeneities for a cardiac ROI, and dynamic pTX shimming using 4 kT-points outperformed static pTx shimming. Increasing the number of kT-points to eight enhanced B_1_^+^ efficiency and homogeneity [112].

A larger number of RF Tx channels introduces some inherent challenges, however. An integral part of RF excitation with a multichannel pTx system is the real-time supervision of SAR on each transmitting channel. Appropriate hardware and software must be implemented to monitor the RF power in real time and terminate RF transmission if any of the pre-settings or regulatory values for local or global SAR are potentially exceeded [113]. Ideally, this capability includes monitoring of the relative phases between channels. The concept of virtual observation points (VOP) has been introduced to reduce the overall complexity and amount of data that needs to be monitored during real-time SAR supervision of a multichannel pTx RF system [114]. Currently, one commercial vendor has announced a clinically certified SAR monitoring system for an 8-channel pTx system based on VOPs [115]. A precondition for the application of the VOP concept is the accurate simulation and verification of each multichannel RF coil to be used [116]. New VOP algorithms are being developed aiming at increasing accuracy and efficiency and reducing potential overestimations to allow for increased RF transmit power while providing SAR safety at the same time [117]. Such algorithms should be particularly advantageous at EHF, where the number of channels in the pTx system is expected to increase and the amount of real-time data may otherwise become unmanageable.

Another significant hurdle regarding pTx systems with high channel count is the necessity to acquire sufficient data to properly calibrate suitable RF pulse waveforms, e.g., maps of the transmit B_1_ field. Ongoing methodological developments at UHF aim at reducing the complexity of exams and the preparation efforts necessary to adjust the pTx imaging parameters for individual patients and body regions. In this context, universal RF pulses have been introduced for UHF imaging of the brain to provide pTx settings that suit most individuals [118–120]. The aim of the universal pulses is to provide pTx settings that are optimized across a set of training individuals and result in adequate flip angle uniformity and safety for a large variety of individuals. This concept saves time otherwise needed to calibrate individual pTx settings for each UHF exam. The concept of universal pulses has recently also been successfully demonstrated for UHF pTx body imaging [121], but it is an open question whether it will ultimately be suitable for body imaging and whether it can be extended to EHF, where RF field pattern complexity and differences between individuals will be even higher. As a further alternative, hybrid calibration methods have also been investigated that combine the concept of universal pulses with a fast subject-specific online optimization (≈ 1 min) to further improve excitation homogeneity [122].

### RF coils for head and body MRI

Besides the magnet, the multichannel pTx system and the associated multichannel RF transmit coils are probably the most critical technical components at UHF and EHF to achieve a successful human imaging platform. The RF coils directly interact with the subject and tissues to be imaged. On the transmit side, they should excite the brain or body region under investigation with high power efficiency, with optimal B_1_ uniformity, and at practical SAR levels to allow for high image quality of the MR experiment and guarantee safety at the same time [123].

Numerous multichannel RF transmit coils have successfully been developed and evaluated for UHF head and brain imaging at 7 T, 9.4 T, and 10.5 T [5, 6, 124], and are also under development for 11.7 T [125]. Simulation work has been pursued to look at coil performance at EHF, for example at 14 T [110, 126] or even as high as 23.5 T (1 GHz) [63]. Additional efforts have also been invested to provide active local B_0_ shimming with additional shimming coils implemented into the design of UHF head coils [98, 127]. At lower field strengths, loop coils dominate for the design of individual elements. Examination of the ideal current patterns at increasing field strengths shows that dipole-like elements become increasingly important to maximize SNR and minimize SAR [128, 129]. This has led to the development of arrays based on a combination of both dipoles and loops, which have been demonstrated experimentally for human imaging up to 10.5 T [5].

Beyond UHF MRI of the brain, imaging in the human body is not as straightforward and requires the development of dedicated multichannel RF transmit arrays. Here, two different concepts have been pursued. First, local RF transmit body array coils have been developed and successfully applied at different field strengths for cardiac or pelvic imaging or in other regions of the human torso, e.g., [130–134]. These local Tx/Rx multichannel RF coils are placed on or close to the body region under investigation and thus resemble the use of surface receiver arrays in conventional clinical MRI systems at lower field strengths. The second strategy for UHF body imaging focuses on the development of integrated multichannel whole-body RF coils that are placed “remotely” behind the bore liner of the MRI system similar to conventional body coils at lower field strengths [109, 135, 136]. This concept requires a higher degree of invasiveness into the structure of the MRI system for body coil integration, but allows for RF transmission using the same transmit coil for any body region with potentially improved RF uniformity, and, furthermore, the limited space in the patient tunnel of the MR system is less obstructed with RF coil components. It also generally depends on the availability of higher transmit power due to lowered B_1_ efficiency and higher channel counts to achieve uniformity [137], but these are primarily engineering and economic challenges rather than fundamental impediments, even at EHF.

### X-nuclei

At high magnetic fields, imaging and spectroscopy with other MR-active nuclei besides ^1^H become increasingly feasible, and multinuclear imaging can provide unique metabolic information [12, 13]. However, the application of multinuclear imaging in clinical research is currently limited due to low SNR, correspondingly large voxel sizes, and long acquisition times. Thus, a 14 T system could tremendously improve the understanding of ion homeostasis and energy metabolism in vivo in humans. In particular, imaging of nuclei with low gyromagnetic ratio, such as ^39^ K and ^35^Cl, will greatly benefit from 14 T. For example, potassium ions (K^+^) play a vital role in myocardial function. While extracellular K^+^ concentrations can easily be estimated from laboratory analysis of blood samples, a method for measuring the intracellular K^+^ content in vivo is greatly needed and would permit novel insights into pathophysiological processes of cardiac diseases, including arrhythmias. Potassium MRI is quite challenging, since the sensitivity is about six orders of magnitude less than that of ^1^H MRI. The sensitivity gain of 14 T will enable, for the first time, quantitative in vivo assessment of the cellular potassium content in the myocardium. This approach opens an entirely new research field of MRI-driven phenotyping as a link to personalized medicine. In the long run, such multinuclear examinations might also enable in vivo imaging of the membrane potential of neurons and other cell types, which is known to be altered in many neurological diseases, such as migraine, epilepsy, and ischemic stroke, but also in myocardial infarction [138].

A key development component of a 14 T imager will thus be multinuclear imaging methodology. Specially tailored acquisition and image reconstruction techniques will have to be implemented to enable efficient imaging of ^19^F, ^23^Na, ^2^H, ^7^Li, ^17^O, ^35^Cl, and ^39^ K, and spectroscopy of ^13^C and ^31^P at 14 T, including methods that address challenges due to B_0_ and B_1_ inhomogeneities and restrictions due to SAR limitations.

For some X-nuclei such as ^19^F, ^31^P, and ^7^Li, the resonant frequency at 14 T is so high and the wavelength in tissue is so short that parallel transmission techniques will be needed similar to ^1^H at 7 T. Even ^23^Na (resonance frequency 158 MHz @ 14 T) and ^13^C (resonance frequency 150 MHz @ 14 T) will benefit from parallel transmission, particularly in the body. Therefore, a large effort will be required to develop the requisite pTx hardware and RF coils, but, in principle, this can be done by leveraging the know-how that has been accumulated for parallel transmission coils for ^1^H at 3 and 7 T.

### Addressing motion

For ^1^H imaging at 14 T, one of the goals is to further increase spatial resolution in many applications. At 7 T, very-high-resolution imaging has been achieved in relatively long scan times with the support of motion tracking and correction methods. Isotropic spatial resolution of a few hundred micrometers has been achieved [25, 139–141]. With such and further improved resolution, the gap between in vivo human imaging, small-animal imaging, and invasive optical imaging can be narrowed. At 14 T, such resolution can be achieved in shorter scan times or resolutions below 100 µm may become possible. This will require sophisticated precise motion tracking and correction, as subject and organ motion will otherwise limit the effective resolution gain. It is to be determined whether external (optical or EM-field mapping) tracking devices, often relying on physically attached markers, or MR-based motion detection by imaging navigators will deliver the required spatial accuracy for this demanding application. Detailed discussion of motion correction requirements, technology, applications, and limitations is beyond the scope of this contribution, but a number of reviews on the topic are available [142–146].

### Conducting implants

A 14 T system will initially be used strictly for research investigations with no benefit to individual participants. In such a scenario, the risk–benefit analysis for inclusion is weighted against including subjects with any kind of implant that may pose a safety risk due to interactions with the electromagnetic fields of the system, in particular either the higher magnetic field or the RF excitation fields. Nonetheless, there is a societal benefit to such research investigations, so that the risk does not have to be reduced to zero (which is also not the case even for those without any implants).

Similar to the developments at 7 T, we expect that exclusion criteria will be very rigorous initially, but may be relaxed as more experience is gained and scientific investigations determine the extent of implant interactions and enable measures to be taken to mitigate any risks. In the early use of 7 T, even dental implants and tattoos that are quite common in the healthy population were excluded at many sites. Research examinations in such subjects were only considered after studies considering their safety [147, 148]. In particular for RF interactions, where the short wavelength at 14 T implies that even small-dimension implants could be resonant, developments in pTx targeting the manipulation of the RF electric fields will likely be key to enabling safe examinations [149, 150]. Such research is also key to ensuring that the benefits of EHF can be applied to a wider segment of the population, as the number of persons with implants has dramatically increased in recent years, and the global market for active implantable devices is, for example, expected to grow at a 7.8% compound annual growth rate between 2022 and 2027 [151]. Thus, unlike the early 7 T situation where implants received minimal attention for many years, implants should be on the agenda of the 14 T project right from the start, with the goal to quickly remove as many of them as possible from the exclusion criteria while ensuring safety.

### Target configuration

Based on the aforementioned considerations, the GUFI consortium has formulated the following target configuration for a 14 T MRI system. These targets may not all be achievable in an initial configuration, but should be considered as a long-term objective:

- A passively shielded, whole-body magnet with an imaging field-of-view of at least 40 cm. Passive shielding is preferred to reduce risk in magnet construction and operation and since the system will presumably be installed at a greenfield site with sufficient area to accommodate a large stray field.
- Whole-body gradients with an imaging field-of-view of at least 40 cm. The performance should match current state-of-the-art. Presently, there are commercial whole-body gradients available that achieve 200 mT/m amplitude and 200 T/m/s slew rate at 3 T [152], but gradients with ≥ 80 mT/m and ≥ 200 T/m/s should be sufficient for many applications. As part of research projects, gradient arrays that enable both imaging and shimming to fourth order will be investigated.
- Dynamic magnetic field shimming capability with capability to accommodate local shim arrays with at least 32 coils [98]. This capability may be extended to 64 or 128 channels, since simulations have shown enhanced shimming performance [153].
- A parallel transmit system with at least 16 channels and 2 kW per channel for the initial applications, particularly in the head. We are unaware of commercial systems that currently go beyond this performance. However, within the GUFI consortium, members have developed their own full pTx system with 32 channels that initially had 1 kW per channel [154], but has since been upgraded to 2 kW per channel. As part of research projects, a wideband system with 64 channels suitable for both ^1^H and X-nuclei is targeted.
- Wideband multinuclear imaging including ^1^H, ^2^H, ^7^Li, ^13^C, ^17^O, ^19^F, ^31^P, ^35^Cl, and ^39^ K, as X-nuclei are considered to harbor significant scientific potential at 14 T.
- A receive chain with at least 128 independent channels suitable for both protons and X-nuclei. A reduced number of 64 would be sufficient for most initial applications.

## Initiatives in planning at 10.5 T and higher

In addition to the German plans to pursue a 14 T whole-body MRI, there are several other initiatives around the world targeted at implementing MRI systems at UHF and EHF field strengths above 10 T. In the UK, funding for an 11.7 T magnet has been announced [155]. The system will be wound from NbTi cooled to 2.2 K [61]. The warm bore of the magnet will be 83 cm, making it capable of accommodating a whole-body gradient set. Similar to the German initiative, it is planned as a national facility (located in Nottingham), providing infrastructure access to researchers from across the UK. The magnet will be passively shielded with approximately 500 tons of iron.

The Institute of Plasma Physics of the Chinese Academy of Sciences has announced a project to design and construct a 14 T MRI magnet with 90 cm warm bore and based on Nb_3_Sn superconducting cables [156]. They have published results regarding components and simulation strategies [156–159]. A different group from the Chinese Academy of Sciences has published the basic design of a whole-body, actively shielded 14 T magnet, as mentioned above [60]. Details of the availability of funding to proceed with the construction of a 14 T system in China or the time schedule for completing the project(s) are unknown to the authors of this article.

Finally, there is also a national initiative to establish a 14 T whole-body MRI system in the Netherlands [160]. Funding for the system was approved in February 2022. The system will be sited in Nijmegen and is centered on a variant of the Neoscan Solutions whole-body magnet design described above [59]. It is the only current magnet design that would be based on HTS. The project is focused on medical science including, for instance, cognitive neuroscience, psychiatric disorders, metabolic diseases, and projects outside the brain.

## Conclusions

The plans for implementation of a 14 T MRI system in Germany operated as an open research facility represent a new level of scientific cooperation between the groups focusing on UHF MR research, similar to the models followed in high-energy physics where it is customary to share resource-intensive infrastructure. Research at 14 T is likely to facilitate fundamental discoveries about the function of the human body, including healthy physiology, disease processes, and aging. Although uncertain without performing the requisite research, a longer term potential exists for translating this field strength into use for clinical diagnostics. Even if the costs of 14 T hinder its wider application for the foreseeable future, the experience at 7 T has demonstrated that technology and findings at UHF often lead to improvements at lower field strengths. As soon as a human EHF MRI magnet is successfully constructed with a superconductor different from NbTi, this would be a fundamental breakthrough for human MRI systems, paving the way for even higher field strengths and at the same time for more accessible MRI at the conventional field strengths.

## Data Availability

No new data are associated with this review article.
